# Assessment of Knowledge, Attitude, and Practice in respect of Medical Waste Management among Healthcare Workers in Clinics

**DOI:** 10.1155/2020/8745472

**Published:** 2020-09-28

**Authors:** Pensiri Akkajit, Husna Romin, Mongkolchai Assawadithalerd

**Affiliations:** ^1^Faculty of Technology and Environment, Prince of Songkla University, Phuket Campus, Phuket 83120, Thailand; ^2^Environmental Research Institute, Chulalongkorn University, Bangkok 10330, Thailand; ^3^Research Program, The Development of Management System for Reduction and Control of Water Contamination and Distribution, Songkhla Lake Basin and the Western Coastline of the South of Thailand, Center of Excellence on Hazardous Substance Management (HSM), Bangkok 10330, Thailand

## Abstract

Medical waste represents a significant health risk and an environmental pollution concern due to its hazardous characteristics. The knowledge and practice of healthcare personnel in respect of the disposal of medical waste is essential to perform effective medical waste management. Therefore, the aim of this study was to assess the knowledge, attitudes, and practices related to medical waste management among healthcare workers in clinics (medical and dental clinics, specialized medical, laboratory clinics, polyclinics, and midwifery clinics) in Phuket, Thailand. A cross-sectional study was designed with stratified-random sampling used to select the sample of 344 respondents from 172 clinics of which data were collected using face-to-face interviews. The results showed that the majority of respondents (87.2%) were female of whom 36.9% were aged (20–29), 52.0% had more than 5 years working experience, and 51.2% had participated in at least one training course regarding medical waste management. The overall scores for knowledge, attitude, and practice were at a high level (89.5%, 91.9%, and 92.2%, respectively). Significant and positive correlations were found between knowledge and attitude (*r* = 0.464), knowledge and practice (*r* = 0.396), and practice and attitude (*r* = 0.519). Statistical analysis using *t* tests and one-way analysis of variance showed that working experience and its duration were significant factors influencing good medical waste management practice. However, local authorities should implement a well-planned collection and transfer process for medical waste in order to reduce the risk of environmental pollution and the risk of infection or injury to healthcare workers and the general public.

## 1. Introduction

Medical waste is generated by healthcare facilities such as hospitals, clinics, blood banks, and laboratories, which may cause infection to any person coming into contact with it. This may consist wholly or partly of human or animal tissue, blood or other body fluids, excretions, drugs or pharmaceutical products, swabs or dressings, syringes, and needles or other sharp instruments. It is waste which unless rendered safe may prove hazardous to any person coming into contact with it [1–3]. Therefore, medical waste can be considered as being of the greatest environmental concern since it can harbor potentially harmful microorganisms and carries the risk of transmission of infections from healthcare facilities to healthcare workers, patients, and general public. In order to prevent harmful consequences to the human health, the community, and the environment, proper medical waste management (MWM) is needed, which entails managing waste from their generation, through separation, collection, transport, and treatment, to their final disposal [4–6]. In many developing countries, MWM is not properly carried out, and there are no clearly defined regulations and a lack of operational standards [7, 8]. It has been reported that the disposal of medical waste mixed with municipal solid waste is likely to occur in clinics due to the small quantity of medical waste generated, the high cost of collection and disposal, and a lack of enforcement from the local authorities [9]. Phuket, the largest island in the southern part of Thailand, is a very famous and popular tourist destination [10]. The number of tourists visiting Phuket is significant, and this affects the amount of waste generation. It has been reported that the amounts of medical waste generated from hospitals and clinics are approximately 1,200 kg/day and 1.32 kg/day, respectively [11]. Phuket's waste generation currently exceeds its capacity for waste disposal, and the island has limited waste management options since the amount of waste generated continually increases. At present, MWM in Phuket is conducted at the hospital level, and the final destination for medical waste is an infectious-materials incinerator for the treatment of medical waste that is regulated by the Thailand Public Health Act and WHO Guidelines and managed by Phuket City Municipality under the supervision of the Board of solid waste management and wastewater of Phuket Province. However, this service is only available for hospitals [12]. Therefore, the mismanagement of medical waste in clinics may represent a significant risk factor for disease transmission even though the amount of infectious waste in clinics is less significant than that from hospitals. However, the lack of appropriate waste management options for clinics can cause a variety of adverse impacts on the communities they serve such as infection transmission and soil and water contamination. Lack of knowledge about waste segregation and waste collection, lack of risk awareness, unsafe waste disposal, and limited financial resources are the key factors that cause the mismanagement of infectious waste. Moreover, healthcare workers are the key personnel responsible for the medical waste management from generation until their final disposal [13]. Therefore, the aim of this study was to assess the knowledge, attitude, and practice (KAP) of MWM among healthcare workers in clinics located in Phuket Province in southern Thailand. The results will provide information regarding the current situation and problems relating to MWM in clinics and will assist the future planning of MWM at Phuket, Thailand.

## 2. Materials and Methods

### 2.1. Study Area and Study Design

**Figure 1:**
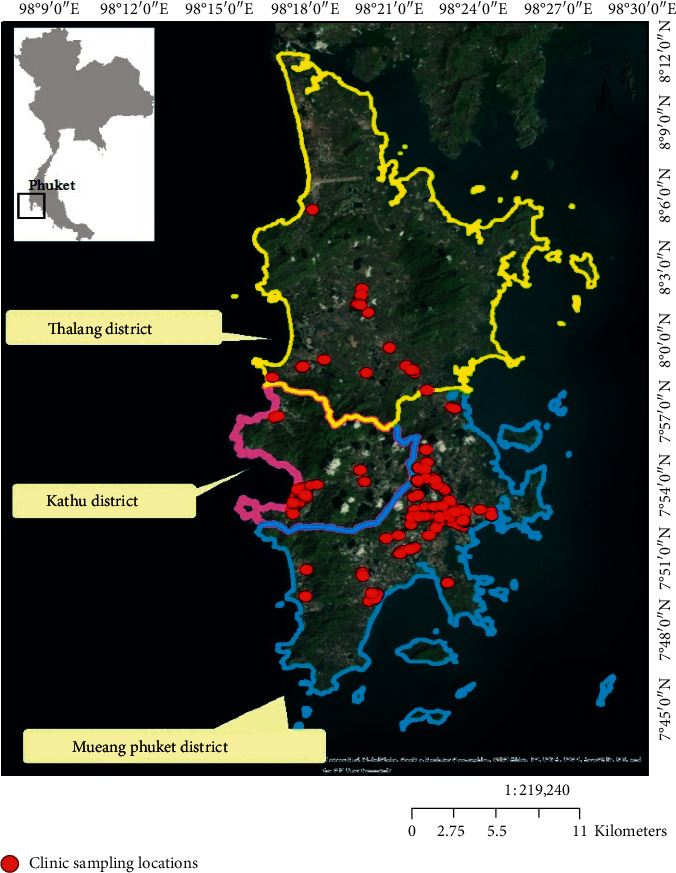
Geographical location of the study area (Phuket Province, Thailand) and the locations of the clinics making up the research sample.

There are three administrative districts in Phuket, namely, Mueang Phuket district, Kathu district, and Thalang district. According to the Bureau of Environmental Health, Department of Health, Ministry of Public Health, Thailand (2017), there are 383 healthcare facilities in Phuket, comprising 6 hospitals, 21 subdistrict health promoting hospitals, and 328 clinics. In this study, the population consisted of all the clinics located in Phuket, except 17 physical therapy and Thai traditional clinics (*N* = 311). The size of the sample was calculated using the Taro Yamane formula [14] with a 95% confidence level. Based on that, the number of clinics included in this study was 172 (*n* = 172). Moreover, a stratified sampling technique was employed to select the type of clinics which made up the sample, which consisted of medical clinics (*n* = 76), dental clinics (*n* = 55), specialized medical clinics (*n* = 32), laboratory clinics (*n* = 6), polyclinics (*n* = 2), and midwifery clinics (*n* = 1). The location of the 172 clinics sampled is illustrated (Figure 1) showing all the sampling locations, which were Mueang Phuket district (*n* = 121), Kathu district (*n* = 33), and Thalang district (*n* = 18). The data were collected by face-to-face interviews with healthcare workers in the 172 clinics between May and July, 2017. Two healthcare workers were selected to be interviewed from each clinic and were those mainly involved in the generation, segregation, and management of medical waste, including doctors, dentists, medical assistants, dentist's assistants, nurses, laboratory scientists, and medical receptionists. Therefore, the total number of respondents in this study was 344 (2 × 172 clinics). The interviews were conducted based on a survey questionnaire, which was designed based on a literature review and the questionnaire was pretested in order to improve the questions. This study was approved by the local Ethics Committee of the Phuket Provincial Public Health Office, Ministry of Public Heath, Thailand. Informed and written consent to participate in the study was obtained by the participants. An explanation of the purpose of research, extent of confidentiality of personal identification, and demographic data were offered to all included participants. The validity and reliability of the section of the questionnaire dealing with knowledge of MWM were tested using the Kuder-Richardson formula 20 (KR-20), while that relating to attitudes and practice in handling medical waste was tested with Cronbach's coefficient alpha [15].

### 2.2. Methods of Measurement

A questionnaire was used in this study to determine the KAP of MWM among healthcare personnel in the selected clinics, which was the primary data with secondary data being obtained from the Bureau of Environmental Health, Department of Health. The questionnaire contained both open-ended and closed-ended items and was divided into five parts as follows: part A: sociodemographic characteristics of the respondents; part B: general information from respondents regarding MWM at clinics; part C: knowledge of respondents about MWM (K); part D: attitudes of respondents towards MWM (A); and part E: practice of respondents in respect of MWM (P). In each part, the items were designed to elicit information about the respondents' KAP relating to four aspects of medical waste: (1) segregation, (2) collection, (3) transportation, and (4) final disposal. Details of the items in the questionnaire used to obtain information about those four aspects were as follows:

1. *Knowledge* of MWM (K) was assessed using 16 items, such as follows: infectious/medical waste and general waste cannot be handled and disposed of together; the quantity of infectious/medical waste in the container or bag should render the bag 1/3 to 2/3 full; and medical waste containers must be closed containers. The knowledge items were scored as either “1” or “0” for the correct or incorrect response, respectively. The total knowledge score for each respondent could range from 0 to 16.
2. *Attitude* towards MWM (A) was assessed using 10 items, such as follows: We consider it necessary to handle medical waste more cautiously. A 3-point Likert scale was used to respond to the items in the attitude section where “agree,” “undecided,” and “disagree” were scored as 2, 1, and 0, respectively. The total attitude score for each respondent could range from 0 to 20.
3. *Practice* in respect of MWM (P) was assessed with 12 questions, such as follows: how often do you separate noninfectious waste from general waste? and how often do you wear gloves while handling infectious/medical waste? The participants were asked to respond to these questions based on a 3-point Likert scale, where “always,” “sometimes,” and “never” were scored as 2, 1, and 0, respectively. The total practice score for each respondent could range from 0 to 24.

### 2.3. Classification of  KAP Scores

The scores of the individual respondents in respect of knowledge about MWM were classified into “high” and “low” categories, while the attitude scores were classified as either “positive” and “negative,” and the practice scores was classified as either “good” or “poor” using the median score for each of the KAP items/questions. The individual knowledge, attitude, and practice scores for each respondent were then aggregated, with high, positive, and good scores being represented by +, while low, negative, and poor scores were represented by -. Then, the aggregated KAP scores were classified into eight possible groups as described by Aluko et al. [16]. The mean score for each item was computed by dividing the overall KAP score from all the respondents by the number of respondents (*n* = 344) and was expressed as percentages. These scores were then categorized into low, medium, and high levels (Table 1).

### 2.4. Statistical Analysis

Statistical analyses were performed to investigate the associations between demographic information and the knowledge, attitude, and practice scores of the personnel in respect of MWM, using independent *t* tests, one-way analysis of variance (ANOVA), and Pearson correlation coefficients. All statistical analyses were conducted using the SPSS version 23.0 software package at a level of significance of 95%.

## 3. Results and Discussion

### 3.1. Part A: Sociodemographic Characteristics of the Respondents

Two healthcare workers from each of the 172 clinics, consisting of one doctor and one staff member, were selected randomly and included in this study. The results revealed that the majority of the 344 respondents were female (87.2%) with the most common age range being 20–29 years old (36.9%). Most of respondents held at least bachelor's degree, and the most common duration of working experience was more than 5 years. The major occupation groups of the respondents comprised medical assistants/nurses/laboratory scientists with experience of handling medical waste in their clinics and had participated in MWM training (Table 2).

### 3.2. Part B: General Information from Respondents regarding MWM at Clinics

**Figure 2:**
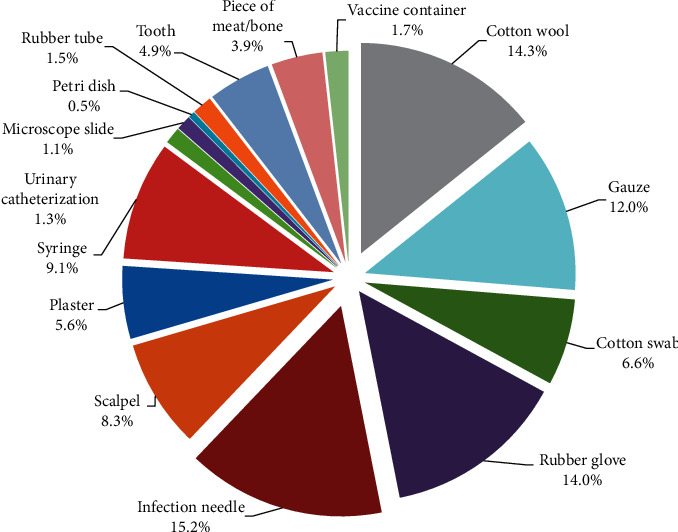
Composition of medical waste from clinics (*n* = 172) in Phuket Province.

The general information about MWM given by the respondents is presented in Table 3. It was found that the majority of the clinics in this study were medical clinics with the amount of medical waste generated reported to be less than 1 kilogram/day. The main categories of medical waste generated in respective clinics were used needles and infectious contaminated cotton wool (Figure 2).

Many respondents generally disposed medical waste into the community's municipal solid waste bin located on a public road while few did not know how to dispose medical waste (Table 3).

From the information obtained, it can be seen that the waste generated in the clinics were not properly handled and could cause the spread of biological agents that represents risk for infection to the community and also the risk to contaminate the environment. The act of disposing own medical waste in the general waste bin, the contents of which would not undergo sufficiently high-temperature incineration to inactivate the microbial content, and insufficient heating at the municipal incinerator may cause further environmental problems due to those pathogens. In addition, there is a chance that scavengers who search for materials with a residual value, such as plastic bottles and cans, in the community's municipal solid waste bin, may suffer accidental injury caused by hazardous needles or sharps if the municipal waste contains medical waste. The results of this study were similar to the findings of Pandit et al. [17] who discovered that most hospitals in Bhopal, India, disposed their waste by open-air burning (83%) and that more than 10% of medical waste was dumped in open fields without pre-treatment. Further, the study of Yong-Chul et al. [18] also showed that medical waste in Korea was often mixed with municipal solid waste, and it is evident that some countries still follow unsafe disposal systems. Thus, appropriate clinical waste management systems should be adopted and implemented to improve MWM.

### 3.3. Part C: Knowledge of Respondents about MWM (K)

It is generally known that healthcare workers' knowledge about MWM is fundamental for proper MWM and is the most important aspect according to Vaught [19]. The respondents' knowledge was categorized into four different groups based on the respondents' occupations (see Table 4). According to the results, a high percentage of the respondents used color coding to identify and classify waste, which indicated a high level of understanding of MWM. Correct responses to the items: Material contaminated with body fluid is medical waste, The color coding for medical waste is red, and Sharp medical waste must be discarded into a hard container were given by 95.3%, 96.5%, and 99.4%, respectively. The results of this study were consistent with those from the study of Abdullah and Al-Mukhtar [20] which found that 79.2% of the respondents at hospitals in Mosul, Iraq used the correct color coding to properly identify medical waste. On the other hand, large numbers of the respondents in this study had incorrect knowledge about the transport and final disposal of medical waste (47.7% and 60.2%, respectively). This contrasted with the study of Singh et al. [21] which found that the majority of doctors (83.3%), paramedics (80%), and medical students (66.7%) at King George's Medical and Dental University, Lucknow, India, had good knowledge about methods of final waste disposal. From the results of this study, facts like there is no medical waste transportation service from clinics to the final disposal destination, and in Phuket, the final disposal of MW is the incinerator operated by Phuket Municipality were answered incorrectly by some doctors/dentists (50.0% and 45.8%, respectively), medical assistants/nurses/laboratory scientists (50.2% and 62.0%, respectively), and others and medical receptionists (70.0% and 61.9%, respectively). There was clearly a lack of knowledge in this area, which could lead to the respondents misunderstanding the correct methods of handling medical waste and could lead to the improper practice of discarding medical waste mixed to community garbage bins in public areas. The results from this part of the study were consistent with those from part B, which showed that 36% of the clinics who participated in this study disposed their medical waste in a public garbage container provided by the municipal waste transportation service. Therefore, relevant authorities and healthcare workers need to follow the municipal waste rules, from the segregation to the disposal processes, in particular, to their transportation and final disposal. The overall scores for the 16 items regarding knowledge of MWM were categorized into high, medium, and low levels according to Rajpal et al. [22], and the outcome is shown in Table 4.

The respondents' high level of knowledge might be because more than half of them had completed at least bachelor's degree (67.7%) and had more than 5 years' work experience. Therefore, long-term employment in clinics may allow personnel to learn more from their working experience which could have contributed to the high level of knowledge about MWM. These findings were consistent with those of Rao et al. [23] who discovered that most respondents (87.8%) in a study conducted at Andhra Medical College, Visakhapatnam, India, had a high level of knowledge about handling medical waste since they had received education about medical waste management from a special agency.

### 3.4. Part D: Attitude of Respondents towards MWM (A)

The scores relating to the attitude of the respondents to MWM are summarized (Table 5). It was found that more than 85% of the respondents had a positive attitude towards MWM, and the overall attitude score based on all the respondents' scores for the 10 attitude items (91.9%) was categorized in the high level. Moreover, the medical receptionists, medical assistants/nurses/laboratory scientists, doctors/dentists, and other occupation groups had overall attitude scores of 95.2%, 93.4%, 85.4%, and 80.0%, respectively.

The responses to the items in the questionnaire showed that the majority of the respondents agreed with the statements, *Medical waste generated in clinics must be handled properly* (97.4%), *Gloves should always be used during medical services to prevent the hazards associated with exposure* (94.2%), and *Medical waste segregation is important* (86.6%). Thus, most respondents paid attention to basic safety precautions in medical waste segregation and agreed that it was necessary to wear gloves to prevent exposure to highly hazardous waste and to control the spread of infection. The self-awareness of healthcare workers in handling medical waste is one of the most important skills that influence the quality of MWM [24]. The study of Nalwaya and Vyas [25] reported that all healthcare personnel (100%) in Saurashtra, India, believed that safe disposal of biomedical waste was their duty and not an extra burden. However, in this study, most (71.5%) but not all the respondents agreed that *Clinical MWM must be more strictly supervised by local government agencies* such as Phuket Municipality. Based on in-depth interviews during the survey, it was found that the majority of respondents would like public health officials to provide training and seminars about MWM related to all aspects, from waste segregation to final disposal. The importance of training regarding MWM must be over emphasized since incomplete or improper knowledge about MWM can have a negative impact on the environment.

### 3.5. Part E: Practice of Respondents in respect of MWM (P)

The respondents' practice in respect of MWM was determined in this study (Table 6), and it was found that 95.6% of the respondents answered *Always* to the question *Do you wash your hands thoroughly after contact with medical waste, even if you wore gloves*? and 93.6% of the respondents answered *Always* to the question *Do you wear rubber gloves during medical service*s? Wearing gloves and hand-washing are widely recognized as practices which are effective in preventing the spread of cross-infection in healthcare facilities [26]. It is suggested that healthcare workers need to pay attention to their own health while giving services to patients. In addition, most of the respondents in this study (95.6%) answered A*lways* to the question *Do you put sharp medical waste into a hard container*? This finding was consistent with Gupta et al.'s finding [27] that most healthcare personnel (more than 80%) only disposed sharps in puncture-proof containers at primary healthcare centres in Lucknow, India. The segregation of needles or other sharp medical waste is important in reducing the risk of accidental punctures or lacerations, which can contribute to the risk of infectious diseases transmission [28].

In relation to the transportation of medical waste, overall, the respondents who answered *Always* to *Do you collect medical waste and take it to a community garbage bin for transportation by Phuket Municipality*? amounted to 42.5% with the medical assistants/nurses/laboratory scientists showing the highest percentage of 45.1% and 41.7%, 40.0%, and 34.9%, respectively, of doctors/dentists, others, and medical receptionists. The incorrect practice of mixing medical waste with community waste represents a risk to the public, especially to scavengers and garbage collectors. Based on the in-depth interviews with the respondents, a lack of understanding of the disposal methods appropriate for medical waste and general waste highlighted a need to improve the knowledge of healthcare personnel in Phuket and thus improve MWM in order to prevent negative impacts to the public and the environment. The results in this part of this study were consistent with those from part C in respect of knowledge about MWM, in which it was found that 47.7% and 60.2% of the respondents, respectively, had incorrect knowledge relating to the transportation and disposal of medical waste. However, the overall practice scores of the healthcare personnel in respect of MWM were at a very high level (92.2%) in respect of the handling of medical waste, which was strikingly similar to the findings relating to their knowledge and attitude of 89.5% and 91.9%, respectively. The findings of this study were, however, not consistent with those of the previous study of Mostafa et al. [15], which reported that the majority of doctors, nurses, and housekeepers at Al-Mansoura University Hospital, Egypt, used inadequate practices relating to MWM, with only 18.9% of the nurses, 7.1% of the housekeepers, and none of the doctors following correct practices. In addition, the study of Ismail et al. [29] found that the MWM practices were poor in all the groups of personnel surveyed at a tertiary healthcare institute in Dakshina, India, and generally, it seems that the prevalence of improper MWM practices in developing countries is alarming due to a lack of proper training and the delegation of the disposal of medical waste to poorly educated workers as discussed by Sapkota et al. [30].

### 3.6. Categorization of the Scores for KAP towards MWM

**Figure 3:**
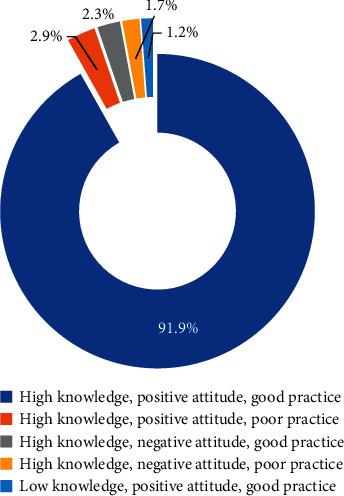
The five composite KAP rating scores.

The respondents were classified further based on their KAP scores using the rating system aforementioned. According to the results, the respondents were included into 5 of the 8 composite rating KAP score categories (Figure 3). The results revealed that the majority of respondents (91.9%) had high knowledge, positive attitude, and good practice (+, +, and +). Only 2.9% of the respondents had high knowledge, positive attitude, and poor practice (+, +, and −), and 2.3% had high knowledge, negative attitude but good practice (+, −, and +) (Figure 3). Therefore, more than 90% of the respondents in the clinics surveyed in Phuket had high/positive/good KAP (+, +, and +) towards MWM which was in contrast to that of the study of Aluko et al. [16], who found that only 38% of healthcare workers in Nigeria had good knowledge, positive attitudes, and good perceptions (+, +, and +), while 20% had poor knowledge, good attitude, and good practice (−, +, and +).

### 3.7. Correlations among Knowledge, Attitudes, and Practice in respect of MWM

Correlation analysis among KAP in respect of MWM was conducted based on Pearson correlation coefficients, and positive and significant correlations (*P* < 0.01) were found between knowledge and attitude (*r* = 0.464), knowledge and practice (*r* = 0.396), and practice and attitude (*r* = 0.519). The moderate correlations recorded in this study can be attributed to the fairly consistently high score levels for the three aspects of the respondents' KAP. It was found that high knowledge of the personnel was associated with positive attitude and good practice towards MWM which was consistent with the classification of the KAP rating score categories, with 91.9% of the respondents showing high knowledge, positive attitude, and good practice (+, +, and +).

No significant differences were detected for the healthcare workers grouped according to their sociodemographic characteristics with the exception that their MWM practice in respect of managing medical waste exhibited significant differences based on the duration of their working experience (*P* < 0.05), and respondents with more experience showed better practices (Table 2). Duration of working experience in healthcare workers is considered to be one of the most significant factors in good practice in respect of MWM, and people with longer working experience tend to have better practical and management skills in respect of MWM than those with less working experience. In this study, it was found that this was particularly the case for those with less than 2 years of working experience.

## 4. Recommendations

There is a need to focus on the control of medical waste disposal and offsite waste transportation to the final disposal destination. Local authorities should provide more training sessions for healthcare personnel who are directly involved in medical waste management in clinics and should also disseminate regulatory information, which will help personnel to understand the issue and perform their jobs properly in compliance with those regulations.

## 5. Limitations of the Study

As this study was confined to only two healthcare personnel from each clinic, more extensive studies with larger population cohort are required for better assessment the KAP in respect of MWM.

## 6. Conclusions

The current study investigated the KAP in respect of MWM of personnel in clinics in Phuket, Thailand, with the aim of contributing information useful in planning for improvements in the MWM system. The high knowledge of healthcare workers was associated with their positive attitude and good practice in respect of MWM and was consistent with their KAP rating scores categorized as high knowledge, positive attitude, and good practice. The duration of working experience of healthcare workers was the most significant factor influencing good practices related to MWM. Illegal waste disposal and the co-disposal of medical waste from clinics with municipal waste could result in a negative impact on people living in communities. However, there is only one infectious-material incineration plant available to clinics and hospitals in Phuket; therefore, investment is needed in providing safer disposal facilities to accommodate all medical waste generated from clinics and hospitals and to guarantee proper safety of the public and prevent the pollution of the environment. Finally, it is vital that policy and regulatory guidelines in respect of medical waste management should be strictly enforced by Phuket Municipality in order to improve MWM practices, especially in the collection and transportation of medical waste.

## Figures and Tables

**Table 1:** Interpretation of the mean KAP item scores in this study.

Questionnaire	Interpretation method
Part A: sociodemographic characteristics	(i) Checklist: a list of items to-tick-off
Part B: general information of the MWM	

Part C: knowledge of MWM	(i) Low if scores <33%
Part D: attitudes towards MWM	(ii) Medium if scores 33–66%
Part E: practice in respect of MWM	(iii) High if scores >66%

Categorization of the scores for KAP	
High K, positive A, and good P	(+, +, +)
Low K, negative A, and poor P	(−, −, −)
High K, positive A, and poor P	(+, +, −)
High K, negative A, and poor P	(+, −, −)
High K, negative A, and good P	(+, −, +)
Low K, positive A, and good P	(−, +, +)
Low K, negative A, and good P	(−, −, +)
Low K, positive A, and poor P	(−, +, −)

**Table 2:** Sociodemographic characteristics of respondents (*n* = 344).

Variables		Frequency (*n*)	Percentage (%)	*P* value
Gender of respondents				
Male		44	12.8	0.342
Female		300	87.2	

Age range of respondents (years)				
<20		6	1.7	0.107
20–29		127	36.9	
30–39		122	35.5	
40–49		66	19.2	
>49		23	6.7	

Educational qualification				
Below bachelor's degree		111	32.3	0.717
Bachelor's degree		192	55.8	
Master's or doctoral degree		41	11.9	

Occupation of respondents			
Doctor/dentist		48	14.0	0.850
Medical assistant/nurse/laboratory scientist		213	61.9	
Medical receptionist		63	18.3	
Others (such as owner, cleaner, and dispenser)		20	5.8	

Years of working experience			
< 2		69	20.1	0.001^*∗*^
2–5		96	27.9	
>5		179	52.0	

Experience in MWM			
Yes		252	73.3	0.009^*∗*^
No		92	26.7	

Training in MWM			
Yes		176	51.2	0.180
No		168	48.8	

^*∗*^Significant 0.05.

**Table 3:** General information about clinical waste management at the respondents' clinic.

Variables	Frequency (*n*)	Percentage (%)
Type of clinic		
Medical clinic	150	43.6
Specialized medical clinic	64	18.6
Dental clinic	111	32.2
Midwifery clinic	3	0.9
Laboratory clinic	12	3.5
Polyclinic	4	1.2

Quantity of medical waste in clinic		
<1 kilogram/day	237	68.9
1–2 kilograms/day	78	22.7
2–3 kilograms/day	9	2.6
3–4 kilograms/day	9	2.6
4–5 kilograms/day	5	1.5
>5 kilograms/day	6	1.7

Medical waste disposal method at clinic		
Do not know	30	8.7
Hire private infectious waste management company	98	28.5
Take to hospital	18	5.2
Take to infectious incinerator myself	74	21.6
Take to general waste bin	124	36.0

**Table 4:** The responses to the items relating to the respondents' knowledge of MWM.

Knowledge of MWM	Doctors/dentists (*n* = 48), *n* (%)	Medical assistants/nurses (*n* = 213), *n* (%)	Medical receptionists (*n* = 63), *n* (%)	Others (*n* = 20), *n* (%)	Total
(1) This is MW					
(1.1) expired medicine					
Correct	35(72.9%)	167(78.4%)	50(79.4%)	17(85.0%)	269(78.2%)
Incorrect	13(27.1%)	46(21.6%)	13(20.6%)	3(15.0%)	75(21.8%)
(1.2) Material contaminated with body fluids					
Correct	48(100.0%)	204(95.8%)	59(93.7%)	17(85.0%)	328(95.3%)
Incorrect	0 (0.0%)	9 (4.2%)	4 (6.3%)	3 (15.0%)	16(4.7%)
(1.3) vaccine container					
Correct	43(89.6%)	170(79.8%)	50(79.4%)	15(75.0%)	278(80.8%)
Incorrect	5(10.4%)	43(20.2%)	13(20.6%)	5(25.0%)	66(19.2%)

(2) Waste generated from healthcare activities is MW					
Correct	44(91.7%)	188(88.3%)	51(81.0%)	8(40.0%)	291(84.6%)
Incorrect	4(8.3%)	25(11.7%)	12(19.0%)	12(60.0%)	53(15.4%)

(3) MW should not be mixed with general waste					
Correct	40(83.3%)	193(90.6%)	57(90.5%)	15(75.0%)	305(88.7%)
Incorrect	8(16.7%)	20(9.4%)	6(9.5%)	5(25.0%)	39(11.3%)

(4) MW should be segregated immediately					
Correct	48(100.0%)	202(94.8%)	57(90.5%)	19(95.0%)	326(94.8%)
Incorrect	0(0.0%)	11(5.2%)	6(9.5%)	1(5.0%)	18(5.2%)

(5) The color coding for MW is red					
Correct	46 (95.8%)	206(96.7%)	60(95.2%)	20(100.0%)	332 (96.5%)
Incorrect	2 (4.2%)	7 (3.3%)	3 (4.8%)	0 (0.0%)	12 (3.5%)

(6) The color coding for general waste is black					
Correct	48(100.0%)	207(97.2%)	61(96.8%)	20(100.0%)	336(97.7%)
Incorrect	0(0.0%)	6(2.8%)	2(3.2%)	0(0.0%)	8(2.3%)

(7) Liquid MW should not be disposed into toilet bowl					
Correct	46(95.8%)	194(91.1%)	58(92.1%)	17(85.0%)	315(91.6%)
Incorrect	2(4.2%)	19(8.9%)	5(7.9%)	3(15.0%)	29(8.4%)

(8) Sharp MW should be separated from other wastes					
Correct	41(85.4%)	184(86.4%)	52(82.5%)	17(85.0%)	294(85.5%)
Incorrect	7(14.6%)	29(13.6%)	11(17.5%)	3(15.0%)	50(14.5%)

(9) MW should be put into a closed container					
Correct	47(97.9%)	207(97.2%)	62(98.4%)	20(100.0%)	336(97.7%)
Incorrect	1(2.1%)	6(2.8%)	1(1.6%)	0(0.0%)	8(2.3%)

(10) Sharp MW must be put into a hard container					
Correct	47(97.9%)	212(99.5%)	63(100.0%)	20(100.0%)	342(99.4%)
Incorrect	1(2.1%)	1(0.5%)	0(0.0%)	0(0.0%)	2(0.6%)

(11) MW container should be filled to no more than three-quarters full					
Correct	40(83.3%)	175(82.2%)	57(90.5%)	10(50.0%)	282(82.0%)
Incorrect	8(16.7%)	38(17.8%)	6(9.5%)	10(50.0%)	62(18.0%)

(12) MW container should be sealed every single day					
Correct	33 (68.8%)	162(76.1%)	53(84.1%)	14(70.0%)	262(76.2%)
Incorrect	15 (31.2%)	51(23.9%)	10(15.9%)	6(30.0%)	82(23.8%)

(13) There is no service for MW transportation from clinics to the final disposal destination					
Correct	24 (50.0%)	106 (49.8%)	36 (57.1%)	14 (70.0%)	180 (52.3%)
Incorrect	24 (50.0%)	107 (50.2%)	27 (42.9%)	6 (30.0%)	164 (47.7%)

(14) In Phuket, the final disposal of MW is the MW incinerator operated by Phuket municipality					
Correct	26 (54.2%)	81 (38.0%)	24 (38.1%)	6 (30.0%)	137 (39.8%)
Incorrect	22 (45.8%)	132 (62.0%)	39 (61.9%)	14 (70.0%)	207 (60.2%)

Categorization of the total scores from all 16 items					
High	41 (85.4%)	193(90.6%)	57(90.5%)	16(80.0%)	308(89.5%)
Medium	6(12.5%)	20(9.4%)	6(9.5%)	4(20.0%)	36(10.5%)
Low	0(0.0%)	0(0.0%)	0(0.0%)	0(0.0%)	0(0.0%)

^∗^MWM: medical waste management; MW: medical waste.

**Table 5:** The responses to the items relating to the respondents attitude towards MWM.

Attitudes towards MWM	Doctors/dentists (*n* = 48), *n* (%)	Medical assistants/nurses (*n* = 213), *n* (%)	Medical receptionists (*n* = 63), *n* (%)	Others (*n* = 20), *n* (%)	Total
(1) MW generated from clinics must be handled properly					
Agree	48 (100.0%)	208(97.7%)	59(93.7%)	20(100.0%)	335(97.4%)
Undecided	0(0.0%)	3(1.4%)	4(6.3%)	0(0.0%)	7(2.0%)
Disagree	0(0.0%)	2(0.9%)	0(0.0%)	0(0.0%)	2(0.6%)

(2) MW segregation is important					
Agree	41(85.4%)	181(85.0%)	57(90.5%)	19 (95.0%)	298(86.6%)
Undecided	2(4.2%)	13(6.1%)	4(6.3%)	0 (0.0%)	19(5.6%)
Disagree	5(10.4%)	19(8.9%)	2(3.2%)	1 (5.0%)	27(7.8%)

(3) Co-disposal of MW with general waste can cause unsafe effects					
Agree	38(79.2%)	171(80.3%)	51(81.0%)	16(80.0%)	276(80.2%)
Undecided	2(4.1%)	4(1.9%)	6(9.5%)	3(15.0%)	15(4.4%)
Disagree	8(16.7%)	38(17.8%)	6(9.5%)	1(5.0%)	53(15.4%)

(4) MW must be collected more carefully					
Agree	39(81.3%)	185(86.9%)	55(87.3%)	15(75.0%)	294(85.5%)
Undecided	2(4.2%)	5(2.3%)	1(1.6%)	0(0.0%)	8(2.3%)
Disagree	7(14.5%)	23(10.8%)	7(11.1%)	5(25.0%)	42(12.2%)

(5) General waste management and MW management are different					
Agree	39(81.3%)	174(81.7%)	52(82.5%)	16(80.0%)	281(81.7%)
Undecided	4(8.3%)	23(10.8%)	8(12.7%)	1(5.0%)	36(10.5%)
Disagree	5(10.4%)	16(7.5%)	3(4.8%)	3(15.0%)	27(7.8%)

(6) Biomedical waste containers should be marked with a biohazard symbol					
Agree	40(83.3%)	178(83.6%)	55(87.3%)	16(80.0%)	289(84.0%)
Undecided	2(4.2%)	9(4.2%)	5(7.9%)	0(0.0%)	16(4.7%)
Disagree	6(12.5%)	26(12.2%)	3(4.8%)	4(20.0%)	39(11.3%)

(7) Gloves should always be used during medical services to prevent the hazards associated with exposure					
Agree	46 (95.8%)	201 (94.4%)	61 (96.8%)	16 (80.0%)	324 (94.2%)
Undecided	2 (4.2%)	4 (1.9%)	2 (3.2%)	2 (10.0%)	10 (2.9%)
Disagree	0 (0.0%)	8 (3.7%)	0 (0%)	2 (10.0%)	10 (2.9%)

(8) MW management in your clinic is proper					
Agree	25(52.1%)	146(68.5%)	50(79.4%)	11(55.0%)	232(67.4%)
Undecided	16(33.3%)	60(28.2%)	13(20.6%)	7(35.0)	96(27.9%)
Disagree	7(14.6%)	7(3.3%)	0(0.0%)	2(10.0%)	16(4.7%)

(9) MW management is your duty					
Agree	42(87.5%)	155(72.8%)	41(65.1%)	16(80.0%)	254(73.8%)
Undecided	5(10.4%)	43(20.2%)	16(25.4%)	1(5.0%)	65(18.9%)
Disagree	1(2.1%)	15(7.0%)	6(9.5%)	3(15.0%)	25(7.3%)

(10) MW management must be more strictly supervised by the local government agencies					
Agree	37(77.1%)	156(73.3%)	37(58.7%)	16(80.0%)	246(71.5%)
Undecided	10(20.8%)	55(25.8%)	24(38.1%)	3(15.0%)	92(26.8%)
Disagree	1(2.1%)	2(0.9%)	2(3.2%)	1(5.0%)	6(1.7%)

Categorization of the total score from all 10 items					
High	41(85.4%)	199(93.4%)	60(95.2%)	16(80.0%)	316(91.9%)
Medium	7(14.6%)	14(6.6%)	3(4.8%)	4(20.0%)	28(8.1%)
Low	0(0.0%)	0(0.0%)	0(0.0%)	0(0.0%)	0(0.0%)

^∗^MWM: medical waste management; MW: medical waste.

**Table 6:** The answers to questions relating to the respondents' practice in respect of MWM.

Practice in respect of MWM	Doctors/dentists (*n* = 48), *n* (%)	Medical assistants/nurses (*n* = 213), *n* (%)	Medical receptionists (*n* = 63), *n* (%)	Others (*n* = 20), *n* (%)	Total
(1) How often do you separate MW from general waste?					
Always	39 (81.3%)	202(94.8%)	58(92.1%)	20(100.0%)	319(92.7%)
Sometimes	2(4.2%)	7(3.3%)	3(4.7%)	0(0.0%)	12(3.5%)
Never	7(14.5%)	4(1.9%)	2(3.2%)	0(0.0%)	13(3.8%)

(2) Do you put general waste into a black container and MW into a red container?					
Always	36(75.0%)	192(90.1%)	51(81.0%)	18(90.0%)	297(86.3%)
Sometimes	4(8.3%)	12(5.7%)	6(9.5%)	1(5.0%)	23(6.7%)
Never	8(16.7%)	9(4.2%)	6(9.5%)	1(5.0%)	24(7.0%)

(3) Do you wear rubber gloves during medical services?					
Always	44(91.7%)	204(95.8%)	54(85.7%)	20(100.0%)	322(93.6%)
Sometimes	4(8.3%)	7(3.3%)	7(11.1%)	0(0.0%)	18(5.2%)
Never	0(0.0%)	2(0.9%)	2(3.2%)	0(0.0%)	4(1.2%)

(4) Do you not put sharp MW into a red plastic bag?					
Always	39(81.3%)	171(80.3%)	45(71.4%)	19(95.0%)	274(79.7%)
Sometimes	5(10.4%)	10(4.7%)	6(9.6%)	0(0.0%)	21(6.1%)
Never	4(8.3%)	32(15.0%)	12(19.0%)	1(5.0%)	49(14.2%)

(5) Do you put sharp MW into a hard container?					
Always	44(91.7%)	205(96.3%)	61(96.8%)	19(95.0%)	329(95.6%)
Sometimes	1(2.0%)	5(2.3%)	2(3.2%)	0(0.0%)	8(2.4%)
Never	3(6.3%)	3(1.4%)	0(0.0%)	1(5.0%)	7(2.0%)

(6) Do you clean spills of liquid MW immediately with proper procedure?					
Always	42(87.5%)	194(91.1%)	56(88.9%)	17(85.0%)	309(89.8%)
Sometimes	0(0.0%)	7(3.3%)	3(4.8%)	0(0.0%)	10(2.9%)
Never	6(12.5%)	12(5.6%)	4(6.3%)	3(15.0%)	25(7.3%)

(7) Do you wear rubber glove when pick up trash that falls on the ground?					
Always	46(95.8%)	178(83.6%)	51(81.0%)	19(95.0%)	294(85.5%)
Sometimes	2(4.2%)	23(10.8%)	9(14.2%)	1(5.0%)	35(10.1%)
Never	0(0.0%)	12(5.6%)	3(4.8%)	0(0.0%)	15(4.4%)

(8) Do you wash your hands thoroughly after contact with MW, even if you had worn gloves?					
Always	48(100.0%)	202(94.8%)	59(93.6%)	20(100.0%)	329(95.6%)
Sometimes	0(0.0%)	4(1.9%)	2(3.2%)	0(0.0%)	6(1.8%)
Never	0(0.0%)	7(3.3%)	2(3.2%)	0(0.0%)	9(2.6%)

(9) Do you close and seal the MW bag when it is 1/3 to 2/3 full?					
Always	18(37.5%)	91(42.7%)	26(41.3%)	5(25.0%)	140(40.7%)
Sometimes	17(35.4%)	45(21.1%)	13(20.6%)	4(20.0%)	79(23.0%)
Never	13(27.1%)	77(36.2%)	24(38.1%)	11(55.0%)	125(36.3%)

(10) Do you not reuse the plastic bag for MW?					
Always	28(58.3%)	131(61.5%)	41(65.1%)	13(65.0%)	213(61.9%)
Sometimes	13(27.1%)	52(24.4%)	14(22.2%)	3(15.0%)	82(23.8%)
Never	7(14.6%)	30(14.1%)	8(12.7%)	4(20.0%)	49(14.3%)

(11) Do you collect MW and take it to a community garbage bin for transportation by Phuket Municipality?					
Always	20(41.7%)	96(45.1%)	22(34.9%)	8(40.0%)	146(42.5%)
Sometimes	6(12.5%)	25(11.7%)	8(12.7%)	1(5.0%)	40(11.6%)
Never	22(45.8%)	92(43.2%)	33(52.4%)	11(55.0%)	158(45.9%)

(12) Do you not flush liquid MW into toilet bowl?					
Always	44(91.7%)	179(84.0%)	55(87.3%)	19(95.0%)	297(86.3%)
Sometimes	0(0.0%)	10(4.7%)	5(7.9%)	1(5.0%)	16(4.7%)
Never	4(8.3%)	24(11.3%)	3(4.8%)	0(0.0%)	31(9.0%)

Categorization of the total score from all 12 questions					
High	43(89.6%)	197(92.5%)	58(92.1%)	19(95.0%)	317(92.2%)
Medium	5(10.4%)	16(7.5%)	4(6.3%)	1(5.0%)	26(7.5%)
Low	0(0.0%)	0(0.0%)	1(1.6%)	0(0.0%)	1(0.3%)

^∗^MWM: medical waste management, MW: medical waste.

## Data Availability

The data used to support the findings of this study are available from the corresponding author upon request.
